# New insights into the role of MADS-box transcription factor gene *CmANR1* on root and shoot development in chrysanthemum (*Chrysanthemum morifolium*)

**DOI:** 10.1186/s12870-021-02860-7

**Published:** 2021-02-06

**Authors:** Cui-Hui Sun, Jia-Hui Wang, Kai-Di Gu, Peng Zhang, Xin-Yi Zhang, Cheng-Shu Zheng, Da-Gang Hu, Fangfang Ma

**Affiliations:** National Key Laboratory of Crop Biology, MOA Key Laboratory of Horticultural Crop Biology and Germplasm Innovation, College of Horticulture Science and Engineering, Shandong Agricultural University, Tai’an, 271018 Shandong China

**Keywords:** CmANR1, MADS-box, Root development, Shoot, Metabolomics, Chrysanthemum

## Abstract

**Background:**

MADS-box transcription factors (TFs) are the key regulators of multiple developmental processes in plants; among them, a chrysanthemum MADS-box TF *CmANR1* has been isolated and described as functioning in root development in response to high nitrate concentration signals. However, how CmANR1 affects root and shoot development remains unclear.

**Results:**

We report that CmANR1 plays a positive role in root system development in chrysanthemum throughout the developmental stages of in vitro tissue cultures. Metabolomics combined with transcriptomics assays show that *CmANR1* promotes robust root system development by facilitating nitrate assimilation, and influencing the metabolic pathways of amino acid, glycolysis, and the tricarboxylic acid cycle (TCA) cycle. Also, we found that the expression levels of TFs associated with the nitrate signaling pathways, such as *AGL8*, *AGL21*, and *LBD29*, are significantly up-regulated in *CmANR1*-transgenic plants relative to the wild-type (WT) control; by contrast, the expression levels of *RHD3-LIKE*, *LBD37*, and *GATA23* were significantly down-regulated. These results suggest that these nitrate signaling associated TFs are involved in *CmANR1*-modulated control of root development. In addition, *CmANR1* also acts as a positive regulator to control shoot growth and development.

**Conclusions:**

These findings provide potential mechanisms of MADS-box TF CmANR1 modulation of root and shoot development, which occurs by regulating a series of nitrate signaling associated TFs, and influencing the metabolic pathways of amino acid and glycolysis, as well as TCA cycle and nitrate assimilation.

**Supplementary Information:**

The online version contains supplementary material available at 10.1186/s12870-021-02860-7.

## Highlight

CmANR1 confers pleiotropic positive effects on both root and shoot development in chrysanthemum.

## Background

Roots are not only essential for water and nutrient uptake, but also for the synthesis of various hormones, organic acids and amino acids in plants [1, 2]. Generally, root growth and development, as well as their responses to changing environmental cues and stressed conditions are usually operated by some complex regulatory networks including molecular components, such as regulatory peptides, small RNAs, and transcription factors (TFs) [3–7]. TFs are particularly crucial for root plasticity and development by regulation of multiple target genes, and their mutation or gain of function might result in dramatic phenotypic modifications responding to the specific conditions [8–10]. MADS-box transcription factors are key regulators of multiple developmental processes in plants [11], but their contributions to root development are rarely uncovered. *ANR1*, a MADS-box transcription factor gene, has been reported to play roles in lateral root development under nitrate-rich situations in several plant species, but so far, its underlying regulatory mechanism of root development has been somewhat restricted to unclear auxin-related biological processes in the nitrate signaling pathway [12]. Moreover, there is little evidence to support its roles in shoot development, in addition to its roles in root development [13, 14]. Therefore, at present, to connect *ANR1* to both root and shoot development has become a new challenge.

Omics analysis has become a widespread tool for identifying relevant biomarkers by measuring the content of biochemicals associated with the action modes at the level of DNA/RNA, metabolites, and proteins [15]. High-throughput transcriptome sequencing has been widely applied for the efficient and comprehensive analysis of molecular mechanisms by discovering differently expressed genes (DEGs) in different organs, tissues and cultivars under some specific conditions [16]. Metabolomics is now increasingly used for identifying potential biomarkers, and exploring networks in organisms under environmental or genetic perturbations [17, 18]. Sometimes, metabolomics combined with transcriptomics or proteomics, referred to as integrated metabolomics, is suited for understanding the metabolism under phenotype of genome function via ‘gene-metabolism-phenotype’ research model [19, 20]. Ultra-high performance liquid chromatography-quadruple time-of-flight mass spectrometry (UPLC-QTOF-MS) and HPLC-ESI have been widely used in plant metabolomic studies. These data provide accurate measurements of vast metabolites and can facilitate the study of complex traits [21–23].

Due to the significant correlation between morphological traits and metabolites in plants, in this study, six samples of *CmANR1*-transgenic and wild-type (WT) (six samples of each) chrysanthemum, respectively, were used for metabolite profiling of roots and shoots. The non-targeted UPLC-QTOFMS-based and GC-MS-based metabolomics analyses were applied to screen for the differently accumulated metabolites in transgenic chrysanthemum relative to its wild type. The metabolomic analysis together with the previous transcriptomic analysis of roots revealed new possible mechanisms underlying the regulation of *CmANR1* on root development in chrysanthemum. Meanwhile, the metabolite profiling results of both roots and shoots of *CmANR1*-transgenic and WT plants indicated that in the *CmANR*1-overexpressing background and *CmANR1*-nitrate signaling dependent condition, there were significant increases of metabolites involved in various amino acid metabolism and carbohydrate digestion and absorption in roots. Meanwhile, the metabolites relevant to amino acid metabolism, such as asparagine and histidine were decreased, and the main metabolites in connection with unsaturated/fatty acids biosynthesis were increased in the shoots of transgenic plants. Moreover, positive effects on shoot development were confirmed in transgenic plants, with a longer shoot and better photosynthetic performance than those of WT plants, suggesting that CmANR1 might exert pleiotropic effects on root and shoot development in chrysanthemum.

## Results

### CmANR1 plays a positive role in root development in chrysanthemum throughout the developmental stage of in vitro tissue cultures

**Fig. 1:**
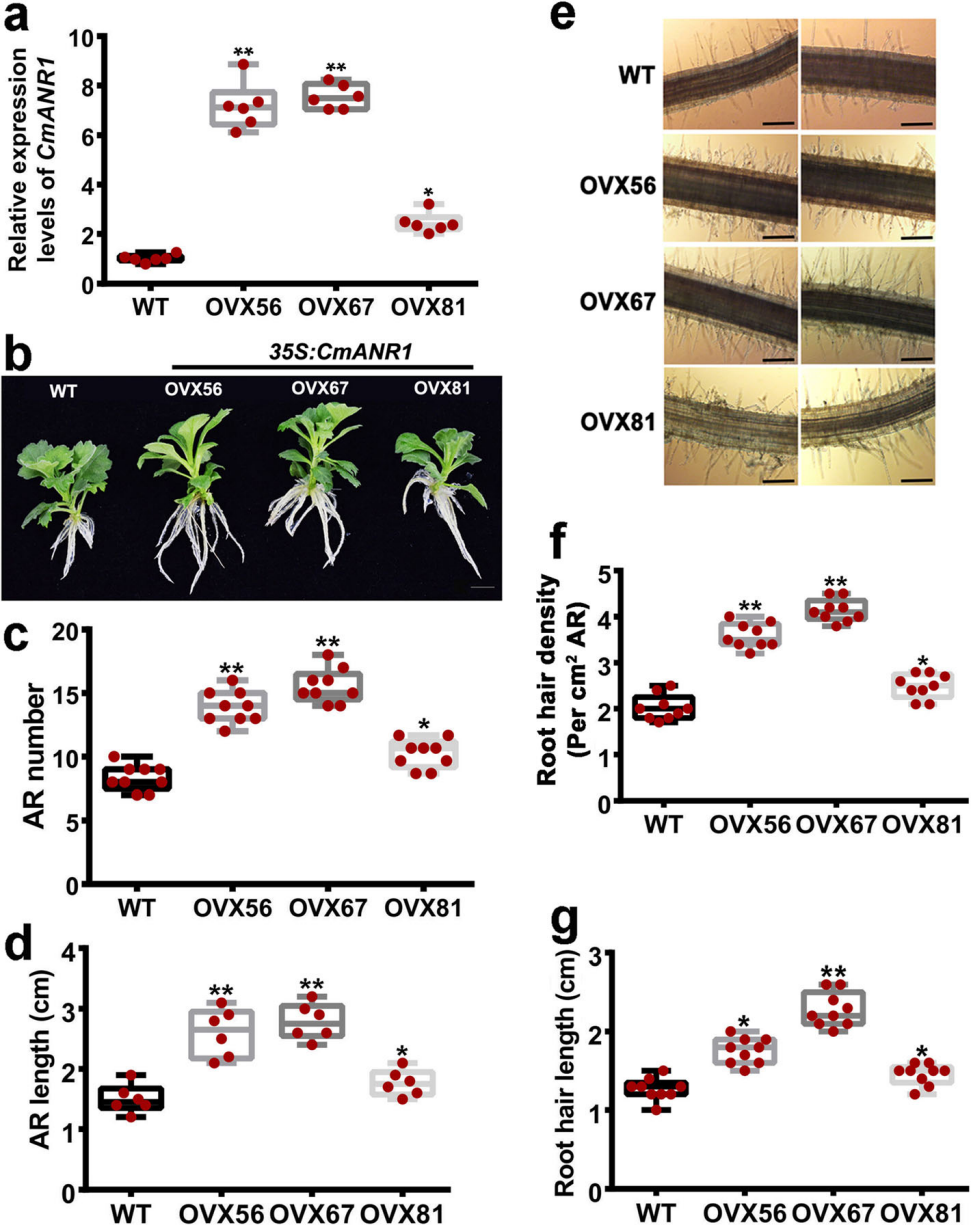
*CmANR1* promotes adventitious root (AR) and hair root development in chrysanthemum. **a** Relative expression of *CmANR1* in three *CmANR1*-overexpressing (OVXs) and wild-type (WT) chrysanthemum lines. **b** Phenotype of *CmANR1*-overexpressing (OVXs) and WT chrysanthemum (10 days-old, in vitro tissue cultures). Scale bar = 1 cm. **c, d** AR number (**c**) and AR length (**d**) of *CmANR1*-OVXs and WT chrysanthemum. **e** Phenotype of root hair on *CmANR1*-OVXs and WT chrysanthemum. Scale bar = 40 μm. **f, g** Root hair density (**f**) and length (**g**) of *CmANR1*-OVXs and WT chrysanthemum. Statistical significance was determined using Student’s *t*-test. No significance (n.s.): *p* > 0.01; *p < 0.01; **p < 0.001

Three independent *CmANR1*-transgenic lines (*CmANR1*-*OVX56,* −*OVX67*, and -*OVX81*) of chrysanthemum were generated by introducing a *35S::CmANR1-GFP* vector into chrysanthemum leaf discs via *Agrobacterium GV3101*, and showing the difference in expression level of *CmANR1* [24] (Fig. 1a)*.* The *CmANR1*-transgenic plants displayed a more forceful growth potency on root development in comparison to the WT plants at the early stage of in vitro tissue culture growth (10 day-olds) (Fig. 1b). The numbers of the adventitious root (AR) in *CmANR1*-OVXs lines were significantly increased by 23.8–69.3% compared to the WT plants (Fig. 1c). Meanwhile, a significant increase in the length of the root was detected in transgenic plants compared to the WT plants, as suggested by the length of ARs (Fig. 1d). Interestingly, light microscope observation showed that the density and length of root hairs in the *CmANR1*-OVXs lines were also obviously increased relative to the WT plants (Fig. 1e-g). These results suggest that CmANR1 positively controls root system development throughout the developmental stage of in vitro tissue cultures.

### Metabolite profiling of roots in both CmANR1-overexpressing and WT chrysanthemum by UHPLC-Q-TOF/MS

The intermediates and its final biochemical products at tissue level are closely related to genotypes, and might directly affect complex quantitative traits in plants [18]. Therefore, identifying the differently accumulated metabolites in roots between transgenic and WT plants might better decipher the underlying regulatory mechanism of *CmANR1* on root development. Thus, the metabolite profiling of roots in both *CmANR1*-overexpressing (*CmANR1*-OVXs) and WT chrysanthemum was tested by ultra-high-performance liquid chromatography-tandem quadrupole/time-of-flight mass spectrometry (UHPLC-Q-TOF/MS). Quality control (QC) samples were regarded as the representative “mean” sample, including all analytes during the analysis and were handled as real samples in the ESI positive or negative analysis batch to monitor the stability of the instrument [25]. Principal component analysis (PCA), orthogonal projections to latent structures discriminant analysis (OPLS-DA), and partial least squares discrimination analysis (PLS-DA) were performed using the retention peaks on all the samples from the study, including conditioning runs and QC samples. All of the QC samples (Blue) were clustered tightly in PCA score plots (Additional file 1). The consistency of the repeated QCs and the reliable data quality of all the experimental samples support the credibility of the method for metabolite profiling in this study.

**Fig. 2:**
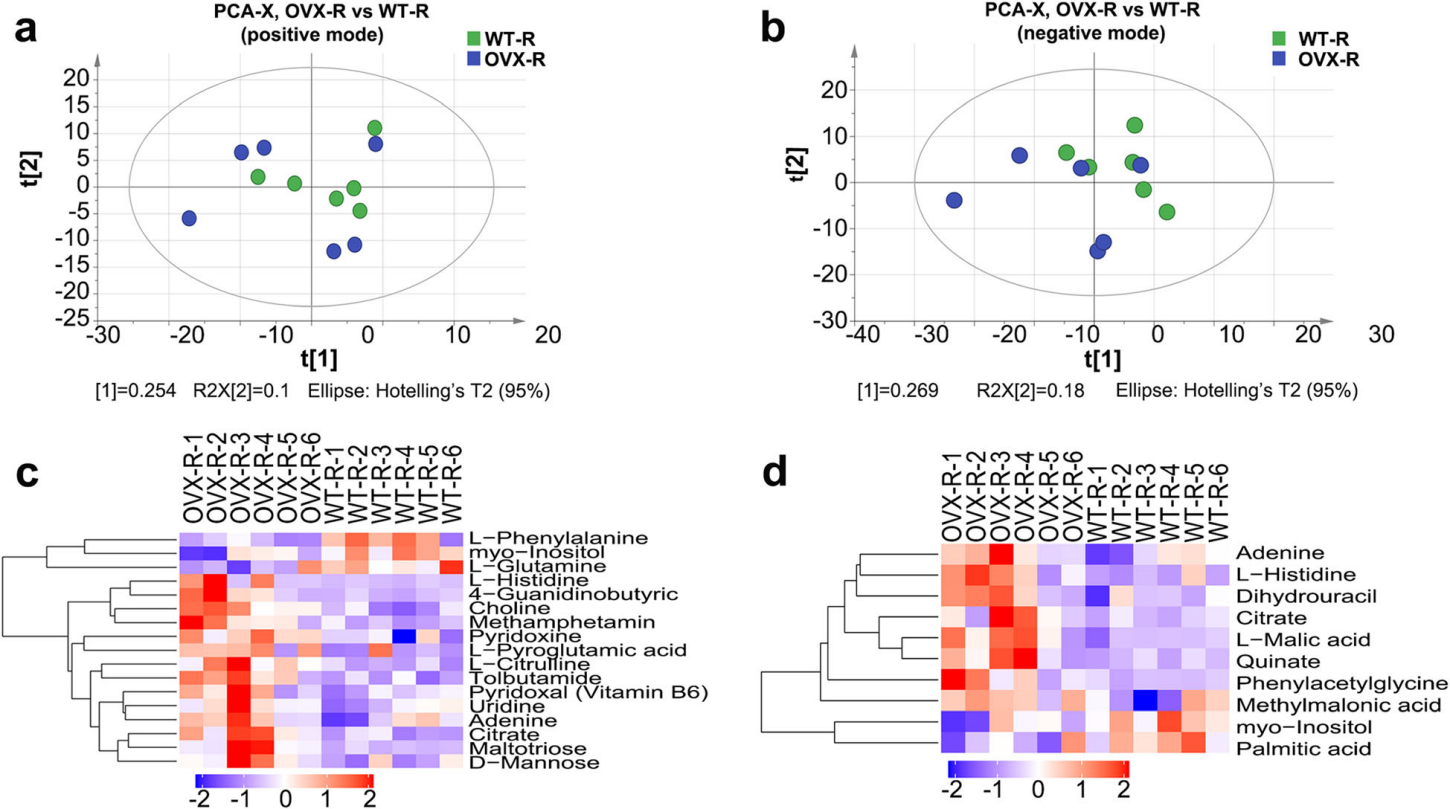
Principal component analysis (PCA) score plots and hierarchical clustering of the metabolites in roots. PCA score plots in ESI positive mode (**a**) and negative mode (**b**) based on HILIC UHPLC-Q-TOF MS data of root samples of *CmANR1*-OVXs and WT chrysanthemum. Hierarchical clustering results of significantly different metabolites of positive (**c**) and negative mode (**d**) data of roots in *CmANR1*-OVXs and WT chrysanthemum

A total of 3194 and 2927 retention peaks, in positive and negative mode, respectively, were extracted from all the samples using XCMS software. PCA score plots in ESI positive mode showed no significant difference, but negative mode revealed a trend of separation between the transgenic and WT samples (Fig. 2a, b). In the OPLS-DA modeling analysis, the evaluation parameter R2Y and Q2 ≥ 0.5, which indicated that the analytical method was robust with good stability and reliability in this study (Additional file 2). Subsequently, variable importance for the projection (VIP) based on the OPLS-DA model could be used to evaluate the expression pattern of each metabolite on the classification discrimination and the explanatory ability of each group of samples, and to explore differential metabolites of biological significance. Using VIP > 1 as the screening standard, differently accumulated metabolites were preliminarily selected in the root samples of *CmANR1*-OVXs and WT chrysanthemum (Table 1). These metabolites were roughly divided into three categories: amino acids, carbohydrates, and alkaloids. Metabolites regarding to various amino acid biosynthesis and metabolism, TCA cycle and pentose metabolism, as well as pyrimidine, vitamin B6 metabolism, were altered when *CmANR1*-OVXs and WT plants were compared (Table 1). Moreover, hierarchical clustering analysis was performed based on the degree of similarity of the significant metabolite abundance profiles in roots. Metabolites with similar expression patterns were clustered together, which indicates their involvement in the relatively close reaction steps of the metabolism processes. For instance, L-histidine and 4-guanidinobutyric acid were clustered together, suggesting their roles in amino acid metabolism; similarly, citrate, maltotriose and D-mannose were closely clustered, indicating their possible actions in glycometabolism in ESI positive mode (Fig. 2c). Meanwhile, organic acid such as L-Malic acid and citrate acid were closely clustered, suggesting their roles in organic acid metabolism in ESI negative mode (Fig. 2d).

**Table 1:** Differently accumulated metabolites in the roots of *CmANR1*-OVXs and WT chrysanthemum

Metabolite name	Content OVXs	Content WT	Fold change (OVXs/WT)	VIP	P-value	Pathway name
**AMINO ACID**						
L-Histidine	4.42E+ 04	1.08E+ 04	4.10	2.39762	0.05473	Histidine metabolism; Aminoacyl-tRNA biosynthesis;
L-Pyroglutamic acid	4.35E+ 05	1.75E+ 05	2.49	6.12216	0.05692	Glutathione metabolism
4-Guanidinobutyric acid	1.65E+ 04	7.65E+ 03	2.16	1.22275	0.03605	Arginine and proline metabolism
L-Citrulline	1.53E+ 04	7.47E+ 03	2.05	1.21049	0.00931	Arginine biosynthesis
Phenylacetylglycine	3.84E+ 04	2.14E+ 04	1.80	1.31318	0.05949	Phenylalanine metabolism
L-Glutamine	1.66E+ 04	2.21E+ 04	0.75	1.06547	0.03932	Arginine biosynthesis; Purine metabolism; Pyrimidine metabolism; Nitrogen metabolism; Alanine, aspartate and glutamate metabolism
L-Phenylalanine	1.52E+ 04	2.82E+ 04	0.54	1.84488	0.01094	Phenylalanine metabolism; Phenylalanine, tyrosine and tryptophan biosynthesis; Tropane, piperidine and pyridine alkaloid biosynthesis
**CARBOHYDRATE**						
Citrate	7.38E+ 03	2.23E+ 03	3.30	1.05757	0.00600	Citrate cycle (TCA cycle)
L-Malic acid	1.03E+ 05	5.26E+ 04	1.96	2.25959	0.01459	Citrate cycle (TCA cycle); Pyruvate metabolism;
Maltotriose	2.07E+ 04	1.19E+ 04	1.74	1.30353	0.03177	Carbohydrate digestion and absorption; ABC transporters
Methylmalonic acid	7.12E+ 04	4.64E+ 04	1.53	1.17634	0.07948	Propanoate metabolism
Quinate	6.42E+ 04	4.19E+ 04	1.53	1.4094	0.03705	Unkown
D-Mannose	1.97E+ 04	1.22E+ 04	1.34	1.21051	0.04827	Fructose and mannose metabolism; Galactose metabolism; Phosphotransferase system (PTS)
Palmitic acid	1.93E+ 05	2.28E+ 05	0.85	2.35796	0.06826	Fatty acid biosynthesis; Fatty acid elongation
**ALKALOIDS**						
Methamphetamine	4.76E+ 04	2.37E+ 04	2.00	1.99384	0.02185	Unkown
Tolbutamide	1.59E+ 04	9.87E+ 03	1.61	1.07417	0.00563	Unkown
Pyridoxal (Vitamin B6)	2.69E+ 04	1.69E+ 04	1.59	1.37275	0.07455	Vitamin B6 metabolism; Vitamin digestion and absorption
Dihydrouracil	8.52E+ 04	5.86E+ 04	1.45	1.38385	0.05778	Pyrimidine metabolism; beta-Alanine metabolism; Pantothenate and CoA biosynthesis
Uridine	2.31E+ 04	1.66E+ 04	1.39	1.06306	0.08419	Pyrimidine metabolism
Adenine	4.37E+ 05	3.41E+ 05	1.38	3.84801	0.07933	Purine metabolism; Zeatin biosynthesis
Pyridoxine	1.40E+ 05	1.09E+ 05	1.29	2.05982	0.01643	Vitamin B6 metabolism
myo-Inositol	4.04E+ 04	5.52E+ 04	0.73	1.94313	0.02757	Inositol phosphate metabolism; Phosphatidylinositol signaling system
**OTHERS**						
Choline	6.82E+ 04	3.14E+ 04	2.17	2.72917	0.00272	Glycine, serine and threonine metabolism; ABC transporters

Note: The differentially accumulated metabolites in roots of *CmANR1*-OVXs were identified by threshold of VIP (Variable Importance in Projection) ≥1 and 0.05 < *P* value< 0.1

### CmANR1 promotes roots system development by facilitating nitrate assimilation

**Fig. 3:**
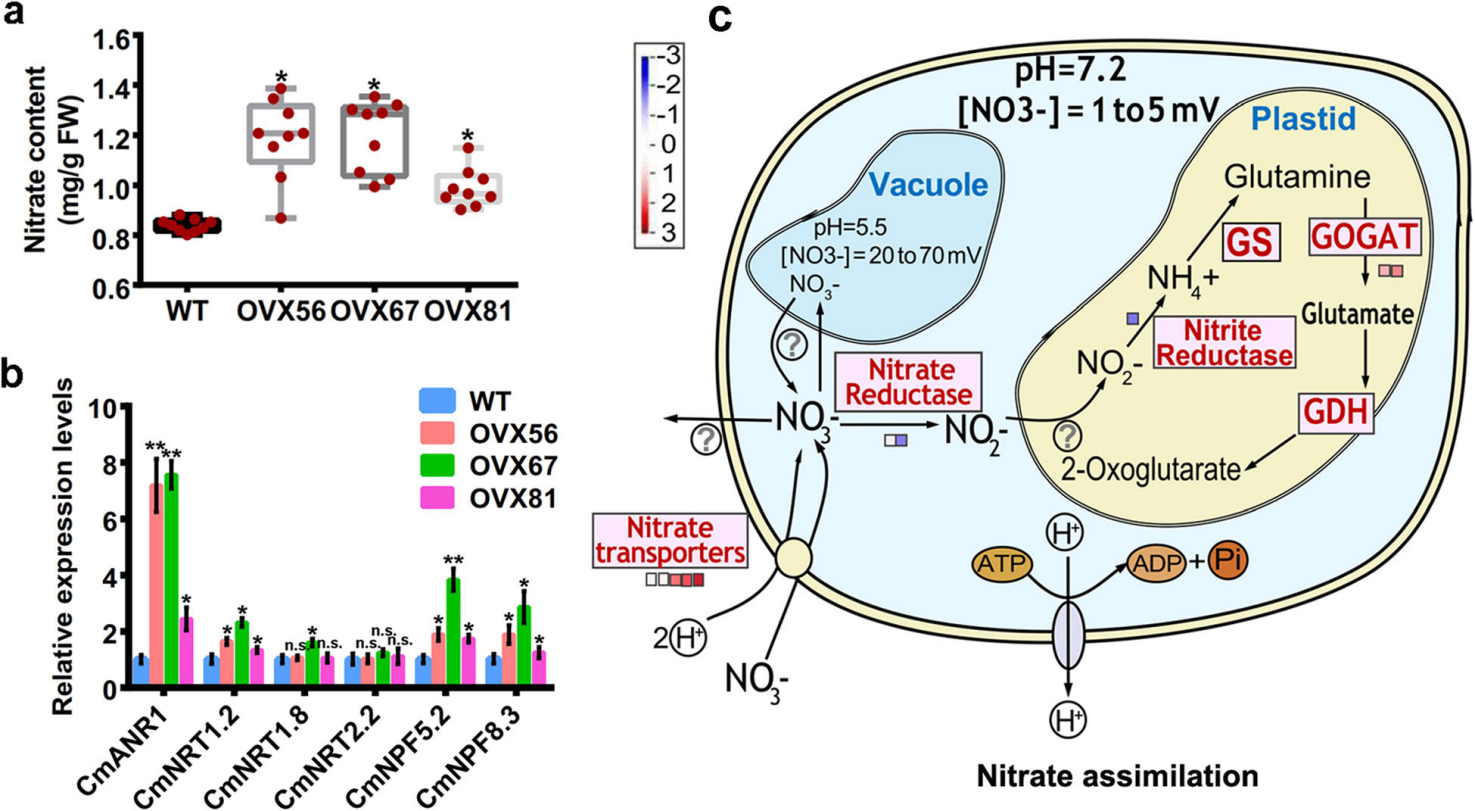
CmANR1 motivates nitrate assimilation in roots of chrysanthemum. **a** Nitrate content in roots of *CmANR1*-transgenic and WT chrysanthemum. **b** Relative expression level of several nitrate/peptide transporter genes in roots of *CmANR1*-transgenic and WT chrysanthemum. **c** The differently expressed genes (DEGs) during nitrate assimilation in roots. Nitrate reductase (TR16877|c0_g8, −0.772337; TR16877|c0_g9, −3.56766), nitrite reductase (TR7757|c0_g2, −1.05013), and *GOGAT* (TR9225|c1_g1, 1.373529) that are involved in nitrate assimilation processes are marked. The red and blue squares mean the upregulated and downregulated genes, respectively. Data are shown as the mean ± SE based on three or more replicates. Statistical significance was determined using Student’s *t*-test. n.s.: *p* > 0.01; *p < 0.01; **p < 0.001

It is well known that nitrate is not only the primary source of nitrogen for most of the higher plants, but it also acts as a signal to regulate global genes expression and many physiological processes in plants [26, 27]. In addition, we have shown that CmANR1 promotes lateral and adventitious roots development in response to higher environmental nitrate concentration signals by altering its trans-activation activity of auxin transport genes in chrysanthemum [28]. Accordingly, the nitrate content in the roots of *CmANR1*-overexpressing chrysanthemum was higher in the WT control, with an increase of 25.5, 27.6, and 13.6% (Fig. 3a). A quantitative real-time PCR (qRT-PCR) assay showed that the expression of several nitrate transporter genes, such as *NRT1.2*, *NPF5.2*, *NPF8.3*, was significantly increased in transgenic plants (Fig. 3b), which can account for increase of nitrate content in the roots of *CmANR1*-OVXs chrysanthemum. Additionally, several genes encoding nitrate reductase (TR16877|c0_g8, − 0.772337; TR16877|c0_g9, − 3.56766), nitrite reductase (TR7757|c0_g2, − 1.05013), and GOGAT (TR9225|c1_g1, 1.373529) that are involved in nitrate assimilation processes showed significantly differential expression in comparison of *CmANR1*-transgenic and WT plants (Fig. 3c). Notably, the root metabolites profiling data showed that several amino acids were differently accumulated in comparison between *CmANR1*-transgenic and WT plants. Four important biomarkers in amino acid metabolism (L-histidine, L-pyroglutamic acid, L-citrulline, and phenylacetylglycine) were increased by 4.10-, 2.49-, 2.05-, and 1.80-fold, respectively, in a ratio of *CmANR1*-OVXs/WT. However, L-glutamine and L-phenylalanine showed changes of 0.75 and 0.54 folds when we compared *CmANR1*-OVXs to WT (Table 1). Once nitrate is transported into cells, it undergoes assimilation and is converted into amnio acids that go on taking part in many physiological processes in roots. Therefore, we propose that CmANR1 promotes roots system development by facilitating nitrate assimilation based on the nitrate content increase, as well as the changes of amino acid content in the roots of transgenic plants.

### CmANR1 influences the metabolic pathways of glycolysis and TCA cycle during root development in chrysanthemum

**Fig. 4:**
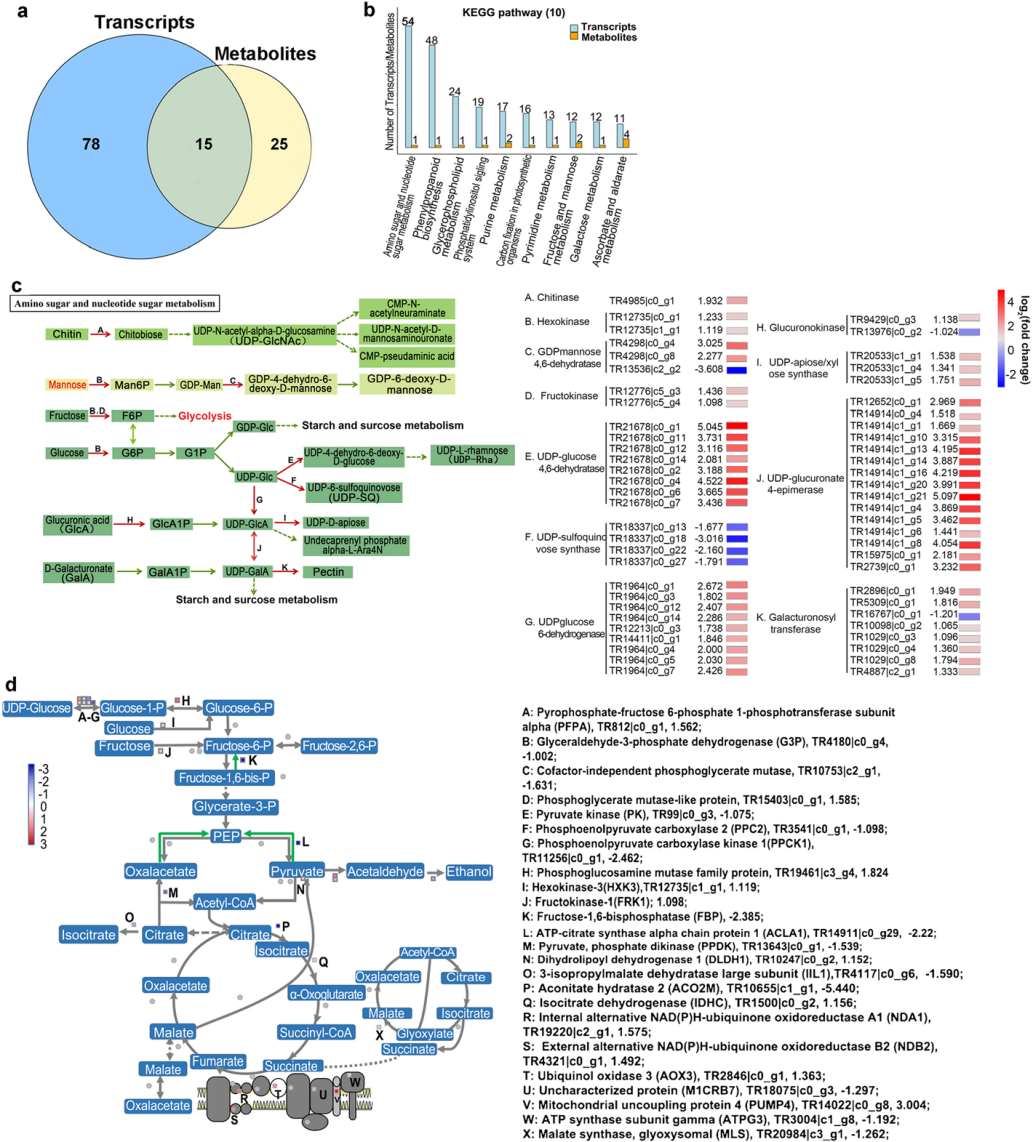
Metabolomic and transcriptomic conjoint analysis of roots in *CmANR1*-trangenic and WT chrysanthemum. **a** Venn diagram of significant differential transcripts and metabolites that were involved in same metabolic pathways in roots of chrysanthemum. **b** The same KEGG pathways (top10) shared by transcriptomic and metabolomic data of roots in chrysanthemum. **c** The representative metabolic pathways and related DEGs that were involved in amino sugar and nucleotide sugar metabolism. Chitinase (TR4985|c0-g1), hexokinase (TR12735|c0-g1), frutokinase (TR12776|c5-g3), and UDP-glucose 4,6-dehydratase (TR21678|c0-g1, TR21678|c0-g4) that are involved in amino sugar and nucleotide sugar metabolism are marked. The red and blue squares mean the upregulated and downregulated genes, respectively. Red arrows suggest where the DEGs function. The log_2_(fold change) and heat meap of the related DEGs in OVXs/WT are listed on the left. **d** The changing metabolites and DEGs being involved in Glycolysis and TCA cycle in *CmANR1*-OVXs chrysanthemum

Our previous transcriptome sequencing was performed with the roots of *CmANR1*-OVXs and WT chrysanthemum, which provided us with much information about the molecular mechanism of *CmANR1* in root development [24]. To further explore more possibilities of the regulatory mechanism of *CmANR1* in roots, we carried out a transcriptomic analysis of roots in *CmANR1*-OVXs and WT chrysanthemum. The Venn diagram showed 36 significant differential transcripts and metabolites were involved in the same metabolic pathway (Fig. 4a). Surprisingly, during the top10 KEGG pathways shared by the two omics, the transcripts involving in ‘Amino sugar and nucleotide sugar metabolism’ were best represented (Fig. 4b; Additional file 4). Then, we listed several representative sugar metabolic pathways in this term and found that the expression of the genes encoding key enzymes in these sugar metabolism pathways was significantly altered in *CmANR1*-transgenic plants. For example, the genes encoding chitinase (TR4985|c0-g1), hexokinase (TR12735|c0-g1), frutokinase (TR12776|c5-g3), and UDP-glucose 4,6-dehydratase (TR21678|c0-g1, TR21678|c0-g4) showed obvious expression differences between *CmANR1*-transgenic and WT plants (Fig. 4c). Moreover, two main metabolites, citrate and malate were significantly accumulated and relative genes were significantly up-regulated and down-regulated during in glycolysis and TCA cycle in the roots of *CmANR1*-OVXs compared to those of WT plants (Fig. 4d), suggesting that CmANR1 might somehow affect glycolysis and the TCA cycle in the roots of transgenic plants. Afterward, these changes during the active biochemical processes might provide more carbon skeletons for the biosynthesis of other metabolites, and more energy equivalents for some physiological processes, facilitating root system development in chrysanthemum.

### Nitrate signaling associated TFs are involved in CmANR1-modulated control of root development

**Fig. 5:**
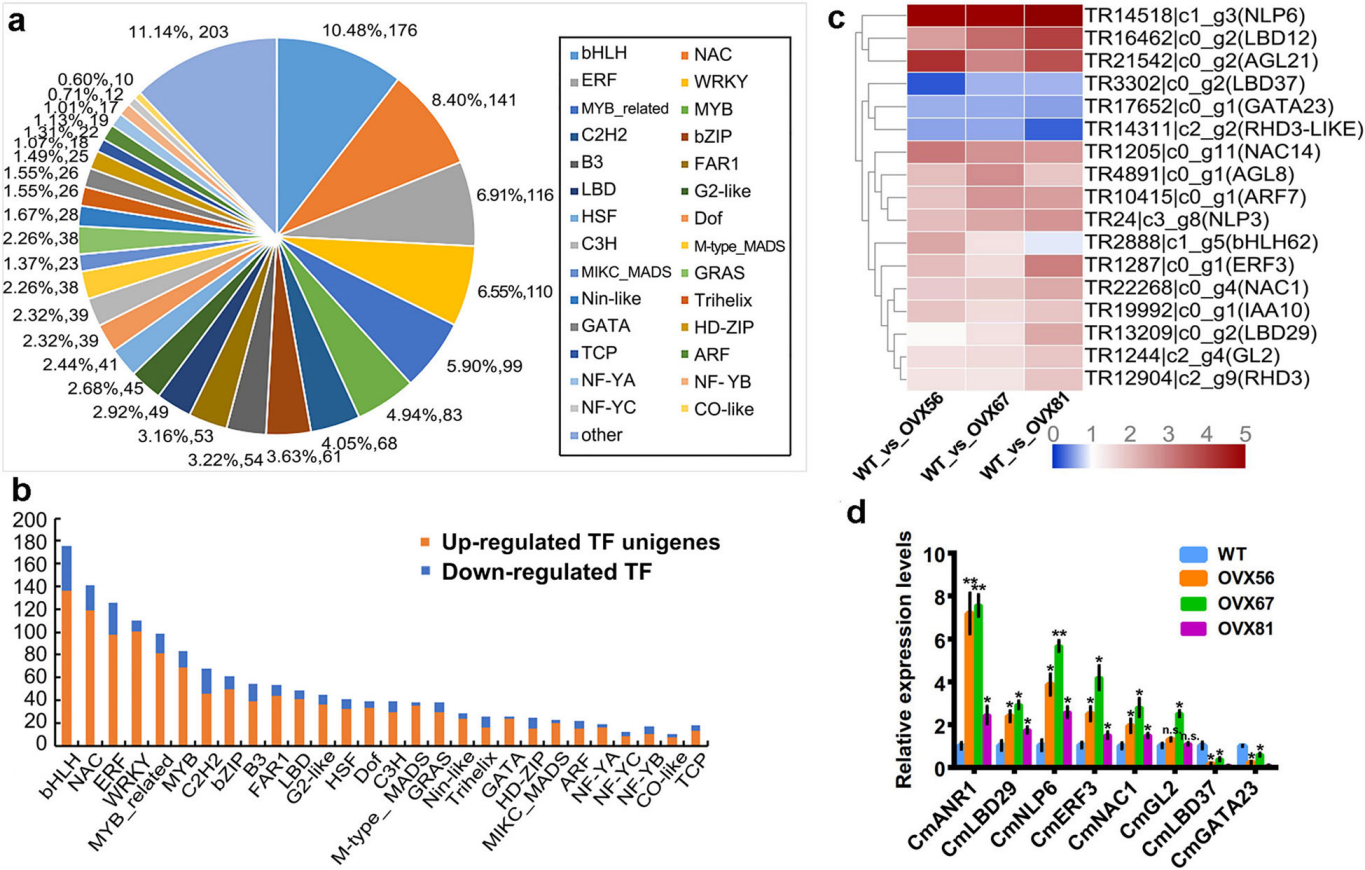
The DEGs annotated as transcription factors (TFs) in the chrysanthemum transcriptome. **a** Number and percentage of TF unigenes of DEGs in chrysanthemum transcriptome. **b** Numbers of up−/down-regulated TF unigenes of DEGs in chrysanthemum transcriptome. **c** The heatmap of TF unigenes related to root/root hair development between *CmANR1*-OVXs and WT. **d** The relative expression level of several TF genes related to root/ root hair development in chrysanthemum. Data are shown as the mean ± SE based on three or more replicates. Statistical significance was determined using Student’s *t*-test. n.s.: *p* > 0.01; *p < 0.01; **p < 0.001

At present, we have clarified two mechanisms, the motivation of nitrate assimilation and possible glycolysis and TCA cycle-related facilitating effect, under the regulation of *CmANR1* on root development in chrysanthemum. However, the exact mechanisms underlying the regulation of *CmANR1* are far more complicated. Transcription factors are important upstream regulators and play critical roles in a wide range of physiological processes in plants. In our previous transcriptome sequencing data, a total of 1752 DEGs encoding inscription factors (TFs) were identified and could be classified into about 34 main different families (Fig. 5a). Apart from a set of very small share families that we named as ‘The other kind’, the largest group of differently expressed transcription factor genes was the bHLH family (176, 10.48%), followed by the NAC (141, 8.40%), ERF (116, 6.91%), WRKY (110, 6.55%), MYB-related (99, 5.90%), MYB (83, 4.94%), and C2H2 (68, 4.05%) families. Notably, the LBD (49, 2.31%), M-type_MADS (38, 2.26%), and TCP (18, 1.07%) families have also a significant proportion (Fig. 5b). Members of these three TF families were deemed to be the most relevant to lateral root development as well as nitrate signaling pathway [11, 29, 30], which was in good accordance with our study background. Then, we selected and drew a heat map of several TF genes from the above-mentioned TF families that have been reported to regulate root or root hair development in other plant species [31–34]. For example, the heat map showed that the expression levels of *AGL8*, *ERF3*, *AGL21*, *LBD29*, and *NLP6* were significantly up-regulated in *CmANR1*-transgenic plants compared to WT plants, while the expressions of *RHD3-LIKE*, *LBD37* and *GATA23* were significantly down-regulated (Fig. 5c). Furthermore, the result of real-time PCR assays of several selected genes related to LR or hair root development was in agreement with the RNA-seq analysis, confirming the validity of the RNA-seq data (Fig. 5d). The expression changes of these genes, on the one hand, suggest their possible correlation with LR or root hair development under the molecular regulation of *CmANR1* in chrysanthemum. On the other hand, the numerous differently expressed TF genes between transgenic and WT plants hint at the complexity of root development under the nitrate treatment conditions.

### CmANR1 acts as a positive regulator to control shoot growth and development in chrysanthemum

**Fig. 6:**
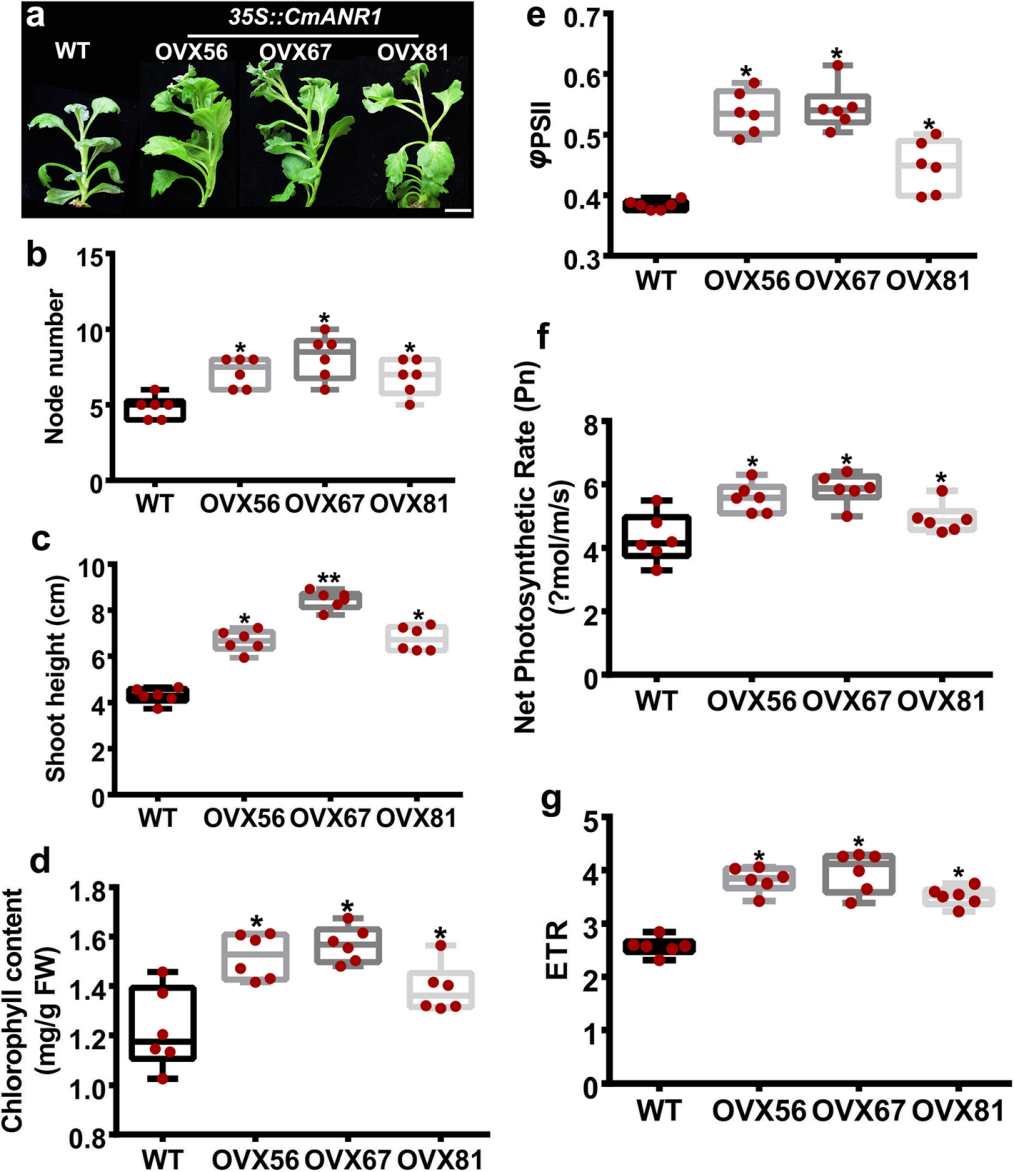
*CmANR1*-overexpressing improves shoot growth and photosynthesis in chrysanthemum. **a** The phenotype of shoots in WT and *CmANR1*-OVXs plants. (20 day-old, in vitro tissue cultures). Scale bar = 1 cm. **b, c** The node number (**b**) and shoot height (**c**) of WT and *CmANR1*-OVXs plants. **d** The total chlorophyll content in mature leaves of transgenic and wild-type chrysanthemum. **e-g** Actual photochemical efficiency (ΦPSII) (**e**), net photosynthetic rate (Pn) (**f**), and electron transfer rate (ETR) (**g**) of leaves of transgenic and wild-type chrysanthemum. The data represent the means ± SE of three independent experiments. Statistical significance was determined using Student’s *t*-test. No(n.s.): *p* > 0.01; *p < 0.01; **p < 0.001

At one time, more concerns were focused on defining the role of *ANR1* in root development, such as its positive regulation of LR initiation and LR length in *Arabidopsis and* chrysanthemum [28, 35, 36], as well as its promotion on LR and PR development in a nitrate-dependent manner in rice [13]. In fact, we found that the shoots of *CmANR1*-transgenic plants exhibited more vigorous growth in the early tissue-cultured period than the shoots of WT plants (Fig. 1a). This phenomenon continued throughout the culture period. We measured the shoot growth of the in vitro tissue-cultured plants. The shoots of transgenic plants were much taller than those of WT plants, with an increase from 55.3 to 97.3% (Fig. 6a). More nodes were observed on shoots of transgenic plants (Fig. 6b), which was a reason for the longer shoot. In addition, the shoot height in *CmANR1*-transgenic plants showed much higher than that of the WT control (Fig. 6c). Also, we carried out detailed studies on total chlorophyll content and photosynthetic properties of leaves on these plants. The total chlorophyll content level in transgenic plants was much higher than that of WT plants (Fig. 6d). And the latter measurements on actual photochemical efficiency (ΦPSII), net photosynthetic rate (Pn), and electron transfer rate (ETR) of the leaves showed that the transgenic plants had significantly higher parameters than those of WT plants, exhibiting a better photosynthetic performance (Fig. 6e-g). The better photosynthetic performance of *CmANR1*-transgenic plants gives a good explanation for their higher and more robust shoot growth.

**Fig. 7:**
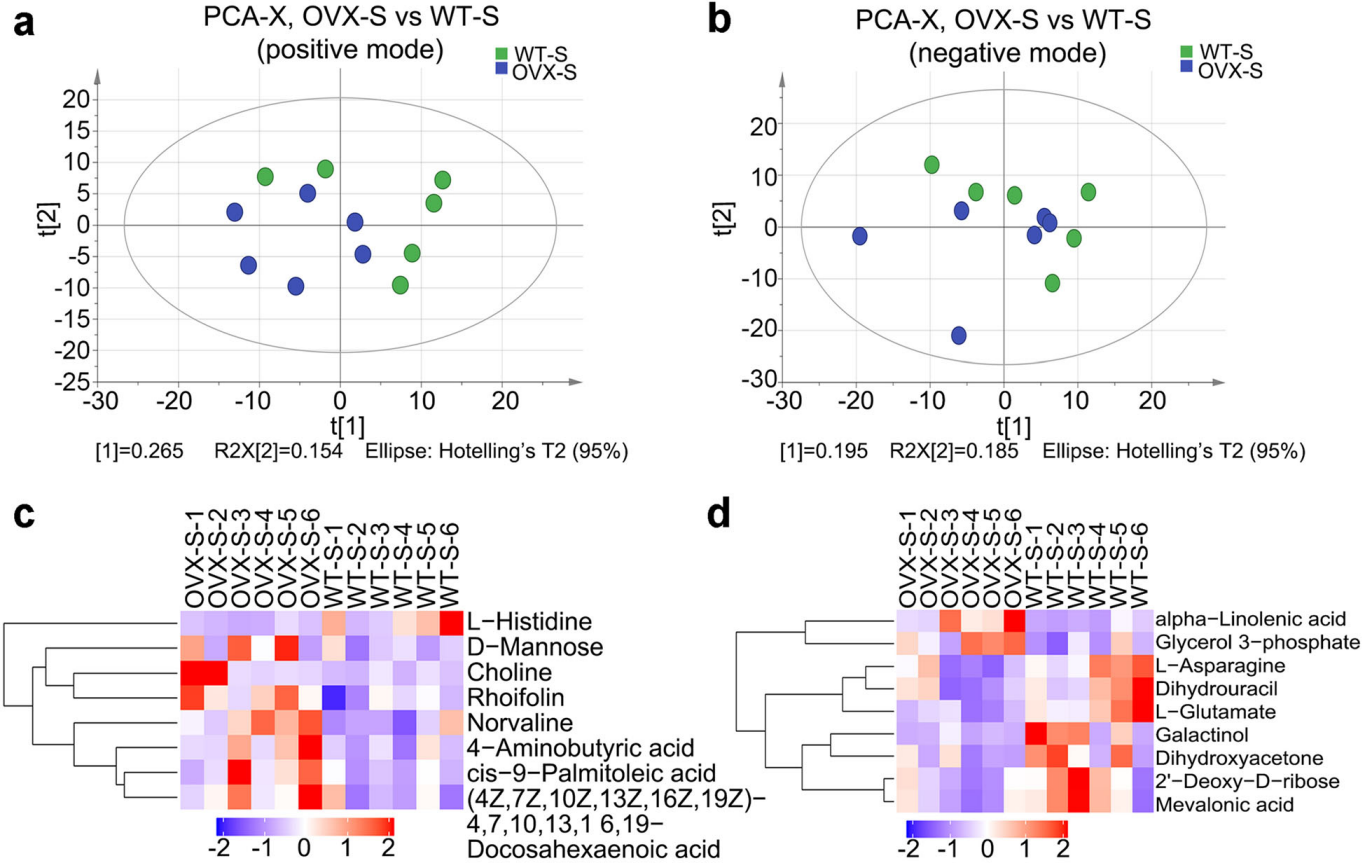
Principal component analysis (PCA) score plots and hierarchical clustering of the metabolites in shoots. PCA score plots in ESI positive mode (**a**) and negative mode (**b**) based on HILIC UHPLC-Q-TOF MS data of shoot samples of *CmANR1*-OVXs and WT chrysanthemum. Hierarchical clustering result of differently accumulated metabolites of positive (**c**) and negative mode (**d**) data of shoots in *CmANR1*-OVXs and WT chrysanthemum

To better explain the shoot developmental differences between transgenic and WT plants, we next evaluated the relative content of the main metabolites by a metabolomics analysis on the shoot parts of two kinds of plants. Similar to the root-metabolomics result, the OPLS-DA score plot and permutation test of both positive mode and negative mode data showed good stability and reliability in this study (Additional file 3). PCA score plots in ESI positive and negative mode based on HILIC UHPLC-Q-TOF MS data revealed a trend of separation between samples (Fig. 7a, b). Hierarchical clustering analysis showed an overview of the content differences and possible connections of the metabolites of transgenic and wild-type shoots that were detected. The heat map and dendrograms showed choline and rhoifolin were clustered together in the positive mode, and both contents were increased, indicating the possible improvement in stress-resistance and activities of secondary metabolites for *CmANR1* transgenic plants. L-glutamate, L-asparagine and dihydrouracil were in closely clustered in the negative mode, which following their possible roles in amino acid metabolism (Fig. 7c, d). Regarding the relative content of the differently accumulated metabolites judged by the fold change in shoots, the situation was distinct from that of roots. Metabolites that were relevant to various amino acids metabolism, like L-glutamate, L-asparagine, and L-histidine were decreased in shoots of transgenic plants. The content of metabolites being involved in fatty or unsaturated fatty acids biosynthesis was significantly increased in shoots of transgenic plants, compared with that of WT plants. However, D-mannose and glycerol 3-phosphate, the main intermediates of nucleotide sugar metabolism and glycolysis, were 2.13- and 1.51-fold increased in shoots of transgenic plants compared with those of WT plants (Table 2). Taken together, we document here that over-expressing *CmANR1* could provoke considerable changes on both root and shoot development in chrysanthemum. *CmANR1* had pleiotropic positive effects on plant growth and development in chrysanthemum.

**Table 2:** Differently accumulated metablites in the shoots of *CmANR1-*OVXs and WT chrysanthemum

Metabolite name	Content (OVXs)	Content (WT)	Fold change (OVXs/WT)	VIP	P-Value	Pathway name
**AMINO ACID**						
4-Aminobutyric acid	2.70E+ 04	1.84E+ 04	1.47	1.02594	0.07333	Alanine, aspartate and glutamate metabolism; Arginine and proline metabolism; cAMP signaling pathway; Nicotinate and nicotinamide metabolism
L-Glutamate	2.20E+ 05	2.84E+ 05	0.77	3.36795	0.00785	Arginine biosynthesis; Alanine, aspartate and glutamate metabolism; Arginine and proline metabolism; Taurine and hypotaurine metabolism; Glutathione metabolism; Nitrogen metabolism......
L-Asparagine	4.70E+ 05	6.89E+ 05	0.68	5.8492	0.02611	Alanine, aspartate and glutamate metabolism; Cyanoamino acid metabolism; Mineral absorption
L-Histidine	8.05E+ 03	5.02E+ 04	0.16	2.38467	0.04143	Histidine metabolism; Aminoacyl-tRNA biosynthesis; Protein digestion and absorption
**CARBOHYDRATE**						
alpha-Linolenic acid	1.64E+ 05	4.06E+ 04	4.04	4.51893	0.02394	alpha-Linolenic acid metabolism; Biosynthesis of unsaturated fatty acids
D-Mannose	5.64E+ 04	2.66E+ 04	2.13	1.84821	0.09906	Fructose and mannose metabolism; Galactose metabolism;Amino sugar and nucleotide sugar metabolism; Phosphotransferase system (PTS)
(4Z,7Z,10Z,13Z, 16Z,19Z)-4,7,10,13,16,19-Docos ahexaenoic acid	4.25E+ 04	2.07E+ 04	2.05	1.65542	0.0736	Biosynthesis of unsaturated fatty acids
cis-9-Palmitoleic acid	2.75E+ 04	1.36E+ 04	2.02	1.29823	0.08320	Fatty acid biosynthesis
Glycerol 3-phosphate	2.88E+ 04	1.90E+ 04	1.51	1.19086	0.02886	Glycerolipid metabolism; ABC transporters
2′-Deoxy-Dribose	1.18E+ 05	2.47E+ 05	0.48	4.45474	0.09508	Pentose phosphate pathway
Galactinol	2.61E+ 03	1.13E+ 04	0.23	1.17585	0.03138	Galactose metabolism
**ALKALOIDS**						
Rhoifolin	6.71E+ 04	5.38E+ 04	1.25	1.28704	0.01828	Flavone and flavonol biosynthesis
Dihydrouracil	9.81E+ 04	1.28E+ 05	0.77	2.08963	0.05783	Pyrimidine metabolism; beta-Alanine metabolism; Pantothenate and CoA biosynthesis
Dihydroxyacetone	2.93E+ 04	4.96E+ 04	0.59	1.77414	0.08589	Glycerolipid metabolism; Galactose metabolism
Mevalonic acid	1.63E+ 04	3.31E+ 04	0.49	1.61854	0.07726	Terpenoid backbone biosynthesis
**OTHERS**						
Choline	2.08E+ 05	4.16E+ 04	4.99	4.30389	0.09518	Glycine, serine and threonine metabolism; Glycerophospholipid metabolism; ABC transporters

Note: The differentially accumulated metabolites in *CmANR1*-OVXs were identified by threshold of VIP (Variable Importance in Projection) ≥1 and 0.05 < P value< 0.1

## Discussion

Based on our previous reports, the current results provide further evidence that *CmANR1* has positive effects on root development in chrysanthemum. The *CmANR1*-transgenic plants have more robust root systems, such as longer and a greater number of LRs, ARs and root hairs, as well as a larger root system surface and volume, in comparison to WT plants (Fig. 1). We have observed and measured the root growth and developmental parameters of several periods of both transgenic and WT chrysanthemum (10-day-old in vitro tissue cultures, 20-day-old in vitro tissue cultures, and 40-day-old in vitro tissue cultures), and it seemed that *CmANR1* could respond quickly to the higher concentration of nitrate (10 mM, under the culture condition) and its positive role in root development was obvious at the initial stage of growth, which might continue into the later growth and development in transgenic plants. On the other hand, in this study, we confirmed that *CmANR1-*transgenic plants grew much higher, were greener, had better photosynthetic performance than those of WT plants under the tissue-cultured conditions (Fig. 6). As *CmANR1-*overexpression in the transgenic plants was triggered by a *35S* promoter in our study, therefore, we could not determine whether the growth advantages of the transgenic plants on the shoot were due to the direct regulation of *CmANR1* on other molecular players in shoots or due to the indirect stimulative effect of more nutrients and water uptake by the more extensive root system of transgenic plants. More work is required to determine the possible mechanism underlying the root-shoot interaction in chrysanthemum.

Previous studies have reported that *ANR1* is a rapid nitrate-responsive and nitrate-dependent transcription factor gene [14, 28]. CmANR1 can act as a transcriptional activator of an auxin transport gene, thereby leading to auxin accumulation in roots during the nitrate signaling pathway in chrysanthemum [24]. Meanwhile, the *CmANR1*-overexpression conferred more nitrate uptake and more nitrate-contained in the roots of the transgenic plants (Fig. 3a). Apart from being as a signaling molecule, nitrate has its basic nutritional role as the main nitrogen source for most land plants, and it will be taken up, translocated, assimilated and transitioned into other physiological compounds (such as amino acids) at the cellular level in the plants [37]. In our root metabolomics assay, it was clear that there were significant increases in the metabolites that were involved in various amino acid metabolisms in transgenic plants, such as L-histidine, L-pyroglutamic acid, 4-guanidinobutyric acid, and L-phenylalanine (Table 1). These findings suggest that *CmANR1* may have some influence on nitrate assimilation during root development under nitrate-rich conditions in chrysanthemum.

The TCA, well-known as an important central pathway connecting almost all metabolic pathways in plants, is responsible for the oxidation of respiratory substrates to drive ATP synthesis [38, 39]. Glycolysis, an oxidization process responsible for the conversion of glucose to pyruvic acid, is another ubiquitous cellular metabolism. Modifications of the expression of important kinases and mutases, such as hexokinase, pyruvate kinase, and phosphoglycerate mutase, could result in dramatic effects on photosynthetic metabolism [40]. Notably, transcriptomics analysis of roots showed DEGs and metabolites related to amino sugar and nucleotide sugar metabolism were mostly enriched. The detailed expression level manifested by fold changes of the genes being involved in glycolysis and TCA cycle were clearly illustrated. The result shows that the expressions of genes encoding hexokinase (TR12735|C1-g1, 1.119), pyruvate kinase (TR99|C0-g3, − 1.075), and phosphoglycerate mutase-like (TR15403|C0-g1, 1.585) were significantly altered in transgenic plants (Fig. 4c, d). Moreover, the significant changes in the content of some respiration substrates, such as citrate, L-malic acid, maltotriose, and D-mannose, suggest a possible energy enhancement in the roots of transgenic plants, which might be another explanation for their better root system development. In conclusion, this evidence hints that there is a link between sugar metabolism and root development that is mediated by *CmANR1*.

Moreover, there were some other interesting metabolites accumulated in transgenic plants. First, choline was one of the most abundant metabolites in both roots and shoots of transgenic plants. Choline, as the primary source for acetyl choline (ACH), is also related to glycine, serine and threonine metabolism, and synthesis of glycine betaine (GlyBet) in the chloroplast [41]. ACH and GlyBet are essential for various important physiological processes and tolerance to abiotic stresses in plants [42, 43] .The increase in the content of choline gave some information on the possible amino acid transition enhancement and stresses-resistance improvement in transgenic plants, which provided a new idea for the future exploration. Second, rhoifolin, as one of the important flavonoids, has various significant biological activities, including anti-allergic, anti-inflammatory, antioxidant, antimicrobial, and anticancer effects [44]. In our shoot metabolomics assay, the content of rhoifolin showed a significant increase in transgenic plants, suggesting the possible improvement in stress-resistance and activities of secondary metabolites for *CmANR1* transgenic plants. Also, some metabolites that were closely related to the biosynthesis of unsaturated fatty acids and fatty acids, such as alpha-Linolenic acid, (4Z, 7Z, 10Z, 13Z, 16Z, 19Z)-4, 7, 10, 13, 16, 19-Docosahexaenoic acid, and cis-9-Palmitoleic acid, were found significantly increased in shoots of transgenic plants, suggesting the improvement of cold and disease resistance in transgenic plants.

**Fig. 8:**
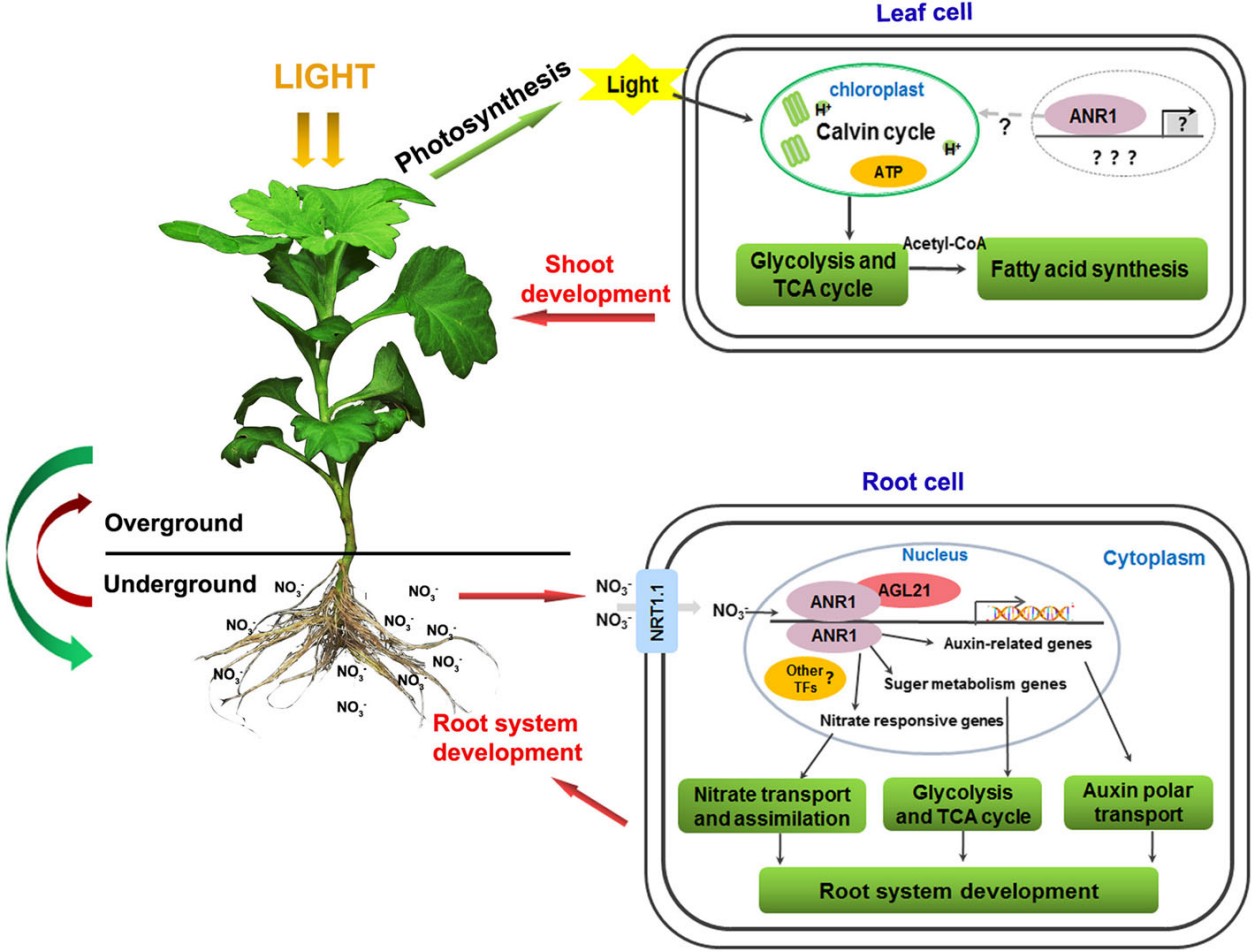
The conclusion of the *CmANR1* working model on both root and shoot development in chrysanthemum

In conclusion, we now present a working model of *CmANR1* on plant development (Fig. 8). In roots: *CmANR1* senses the higher nitrate signal passed by the sensor NRT1.1, which is located on the plasma membrane in roots. Then, CmANR1 formed a homodimer with another CmANR1, and a heterodimer with AGL21, trans-activating downstream target genes, such as auxin-related genes [24], sugar metabolism genes and nitrate responsive genes, resulting in changes in the related physiological processes in plants. The final increases of auxin [24], AAs, metabolites related to glycolysis, and TCA cycle in roots (Table 1) promoted root system development in chrysanthemum. Moreover, TF expression analysis suggested some TFs, such as NLPs, LBDs, and other MADS-box TFs, were more likely to act together with CmANR1 on root system development in this background (Fig. 5). Therefore, it can be concluded that *CmANR1* promoted root system development in chrysanthemum by combining the nitrate signaling pathway with auxin transport, nitrate assimilation, as well as glycolysis and the TCA cycle.

In shoots, the significant increases in shoot height, chlorophyll content, and three photosynthesis parameters (Pn, ETR, and ΦPSII) in transgenic plants indicated a more robust growth and better photosynthesis performance in the shoots of transgenic plants (Fig. 6). However, whether *CmANR1* directly exerted some effects on shoot development could not yet be determined. At the minimum, overexpression of *CmANR1* could trigger shoot development. Taken together, over-expressing *CmANR1* could prompt both root and shoot development in chrysanthemum.

## Conclusion

In this study, we report that CmANR1 plays a positive role in root system development in chrysanthemum throughout the developmental stages of in vitro tissue cultures. Metabolomics combined with transcriptomics assays show that CmANR1 promotes robust root system development by facilitating nitrate assimilation, and influencing the metabolic pathways of amino acid, glycolysis, and the tricarboxylic acid cycle (TCA) cycle. Also, we found that the expression levels of TFs associated with the nitrate signaling pathways, such as *AGL8*, *AGL21*, and *LBD29*, are significantly up-regulated in *CmANR1*-transgenic plants relative to the wild-type (WT) control; by contrast, the expression levels of *RHD3-LIKE*, *LBD37*, and *GATA23* were significantly down-regulated. These results suggest that these nitrate signaling associated TFs are involved in CmANR1-modulated control of root development. In addition, CmANR1 also acts as a positive regulator to control shoot growth and development. These findings provide potential mechanisms of MADS-box TF CmANR1 modulation of root and shoot development, which occurs by regulating a series of nitrate signaling associated TFs, and influencing the metabolic pathways of amino acid and glycolysis, as well as TCA cycle and nitrate assimilation.

## Methods

### Plant materials and growth conditions

The wild-type (WT) tissue cultured chrysanthemum were kindly provided by Professor Junping Gao (China Agricultural University). Dr. Cui-Hui Sun undertook the formal identification of the *CmANR1*-transgenic chrysanthemum used in this study. Three independent *CmANR1*-transgenic lines (*CmANR1*-*OVX56,* −*OVX67*, and -*OVX81*) of chrysanthemum were generated by introducing a *35S::CmANR1-GFP* vector into chrysanthemum leaf discs via *Agrobacterium GV3101* [24]. *CmANR1*-transgenic and WT chrysanthemum were cultivated in vitro on Murashige and Skoog (MS) medium in a standardized culture room of Shandong Agricultural University. These materials have been deposited and publicly available in herbarium of Shandong Agricultural University. The deposit number is SDAU510008.

### Morphological characterization of roots in chrysanthemum

About 10-day-old in vitro tissue-cultured chrysanthemum plants were applied for root morphological characterization. Phenotype pictures of the root hairs on *CmANR1*-OVXs and WT chrysanthemum were photoed under optical microscope (Olympus CX31, Japan). Both the adventitious root (AR) number and root hair density of the plant materials were measured using Image J software (NIH, Bethesda, MD, USA). The length of AR and root hair were calculated by the R Project Program (GNU, New Zealand).

### RNA-Seq analysis

The high-quality RNA extraction for the roots of 10-day old transgenic and WT chrysanthemum (three biological replicates of each sample), library conduction, and RNA-Seq performed by the Illumina HiSeq2000 were carried out professionally by the Ori-gene Biotechnology Company (Ori-gene Inc., Beijing, China). The RNA-Seq data processing, de novo assembly, and annotation were carried out as the previously described [45, 46]. These RNA-Seq sequencing data refered in this study were deposited in the NCBI SRA database (Accession to cite for these SRA data: PRJNA692061; the website link: https://www.ncbi.nlm.nih.gov/sra/PRJNA692061).

### UHPLC/Q-TOF settings

*CmANR1*-transgenic and WT chrysanthemum (six biological replicates of each sample) were used for metabolite profiling of roots and shoots. For the metabolomics and mass spectrometer (MS), as well as the metabolomics data processing and analysis were performed as the previously described [22, 47].

### Quantitative real-time PCR (RT-qPCR)

Total RNAs were extracted from roots of *CmANR1*-transgenic and WT chrysanthemum using TRIzol Reagent (Vazyme, Nanjing, China), followed by reverse transcription (RT) using a PrimeScript first-strand cDNA synthesis kit (TaKaRa, Dalian, China). The qPCR assay reaction procedure and calculation method were referred to as Hu et al [48]. The primers used for qPCR assays are listed in Additional file 5.

### Total chlorophyll analysis

Fresh leaves (~ 0.2 g) of chrysanthemum were cut into small pieces and ground using a little quartz sand and 2–3 mL 96% ethanol (v/v) until completely white. The chlorophyll-containing liquid was filtered and extracted in a total of 25 mL 96% ethanol. The extraction was measured using an ultraviolet spectrophotometer (U-5100, Hitachi, Japan) at A_649_, A_665_, and A_470_. The chlorophyll contents were calculated according to the following formula: Chla = 13.95A_665_–6.88A_645_; Chlb = 24.96A_649_–7.32A_665_. The detailed operation procedure was referred to Hu et al [49].

### Chlorophyll fluorescence parameters

Chlorophyll fluorescence was measured using a FMS-II plus modulated fluorometer (Hansatech, United Kingdom) on the same leaves from 10:00 AM to 1:00 PM. Minimal fluorescence (F_0_), maximal fluorescence (F_m_), PSII (determined as F_v_/F_m_), light-adapted maximum fluorescence (F_m_′), and steady-state fluorescence yield (F_s_) were measured and calculated referring’ to the previous studies [50, 51]. The quantum efficiency of PSII (ΦPSII) was determined as (Fm′- Fs)/Fm′ [52].

### Statistical analysis

All samples were biological analyzed at least in triplicate and represented as the mean ± standard deviation unless specifically labeled. Significance analysis was performed using Student’s *t* test. *P*-values ≤0.01 were considered to be significant, *p*-values ≤0.001 represented a very significant difference, and n.s. indicates no significant difference.

## Supplementary Information

**Additional file 1: Fig. S1.** PCA score plots in ESI positive mode and negative mode of the root and leaf samples of *CmANR1*-OVXs and WT chrysanthemum.

**Additional file 2: Fig. S2.** Orthogonal partial least squares discriminant analysis (OPLS-DA) of metabolites in roots of *CmANR1*-OVXs and WT chrysanthemum.

**Additional file 3: Fig. S3.** Orthogonal partial least squares discriminant analysis (OPLS-DA) of metabolites in shoots of *CmANR1*-OVXs and WT chrysanthemum.

**Additional file 4: Table S1.** Significantly enriched KEGG pathways between *CmANR1*-OVXs and WT chrysanthemum.

**Additional file 5: Table S2.** The primers used in this study.

## Data Availability

The datasets supporting the conclusions of this article are included within the article and its additional files. The *CmANR1* sequence is available at chrysanthemum genome database (http://www.amwayabrc.com/). *CmANR1*-transgenic and WT chrysanthemum (ID: SDAU510008) were deposited and publicly available in herbarium of Shandong Agricultural University. The RNA-Seq sequencing data refered in this study was deposited in the NCBI SRA database (Accession to cite for these SRA data: PRJNA692061; the website link: https://www.ncbi.nlm.nih.gov/sra/PRJNA692061).

## Abbreviations

WT: Wild-type
AR: Adventitious root
F_0_: Minimal fluorescence
F_m_: Maximal fluorescence
F_v_/F_m_: PSII
F_m_′: Light-adapted maximum fluorescence
F_s_: Steady-state fluorescence yield
