# Supplementary material for: New insights into the role of MADS-box transcription factor gene CmANR1 on root and shoot development in chrysanthemum (Chrysanthemum morifolium)
Source: BMC Plant Biol. 2021 Feb 6;21:79. doi: 10.1186/s12870-021-02860-7 (PMC7866475; doi:10.1186/s12870-021-02860-7)
Supplement: Supplementary file 1 — Additional file 1: Fig. S1. PCA score plots in ESI positive mode and negative mode of the root and leaf samples of CmANR1-OVXs and WT chrysanthemum. [file 12870_2021_2860_MOESM1_ESM.docx]

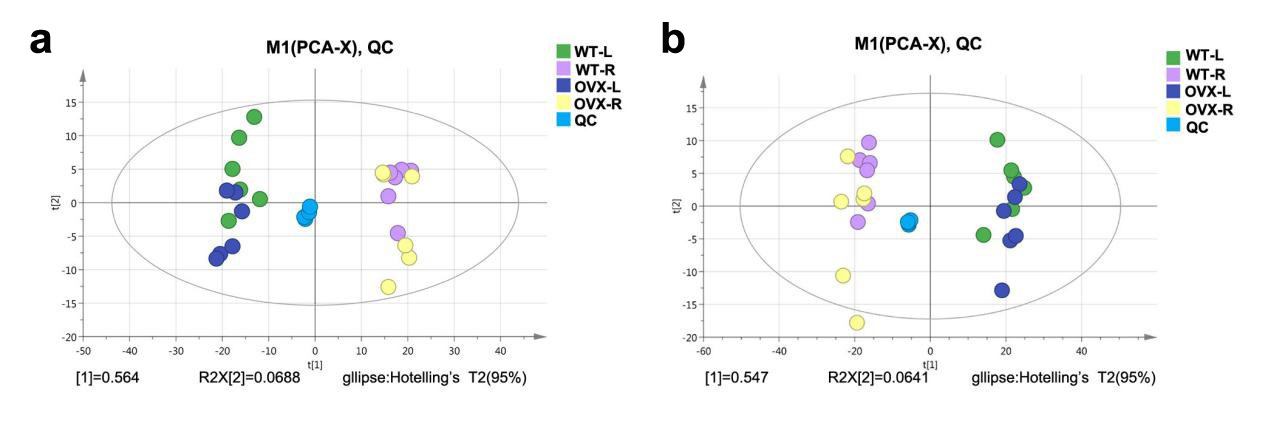


**Fig. S1** PCA score plots in ESI positive mode (**a**) and negative mode (**b**) based on the UHPLC-Q-TOF MS data of the root and leaf samples of *CmANR1*-overexpressing (OVXs) and WT chrysanthemum.
