# Supplementary material for: New insights into the role of MADS-box transcription factor gene CmANR1 on root and shoot development in chrysanthemum (Chrysanthemum morifolium)
Source: BMC Plant Biol. 2021 Feb 6;21:79. doi: 10.1186/s12870-021-02860-7 (PMC7866475; doi:10.1186/s12870-021-02860-7)
Supplement: Supplementary file 3 — Additional file 3: Fig. S3. Orthogonal partial least squares discriminant analysis (OPLS-DA) of metabolites in shoots of CmANR1-OVXs and WT chrysanthemum. [file 12870_2021_2860_MOESM3_ESM.docx]

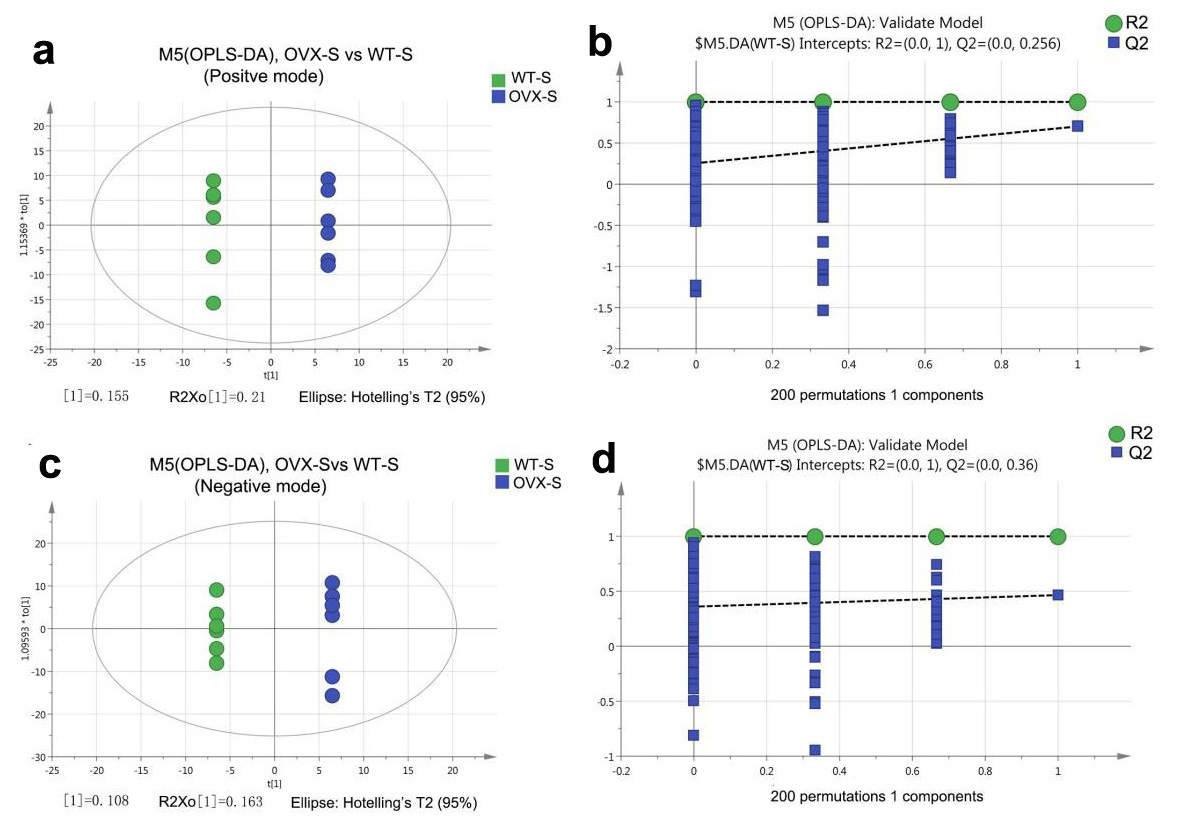


**Fig. S3** Orthogonal partial least squares discriminant analysis (OPLS-DA) of metabolites in shoots of *CmANR1*-OVXs and WT chrysanthemum. (**a**) The OPLS-DA score plot and permutation test (**b**) of positive mode data. (**c**) The OPLS-DA score plot and permutation test (**d**) of negative mode data. R2Y and Q2≥ 0.5 represented a good stability and reliability of the model.
