# Supplementary material for: New insights into the role of MADS-box transcription factor gene CmANR1 on root and shoot development in chrysanthemum (Chrysanthemum morifolium)
Source: BMC Plant Biol. 2021 Feb 6;21:79. doi: 10.1186/s12870-021-02860-7 (PMC7866475; doi:10.1186/s12870-021-02860-7)
Supplement: Supplementary file 4 — Additional file 4: Table S1. Significantly enriched KEGG pathways between CmANR1-OVXs and WT chrysanthemum. [file 12870_2021_2860_MOESM4_ESM.docx]

**Table S1** Significantly enriched KEGG pathways between *CmANR1*-OVXs and WT chrysanthemum (In Root).

| **Pathway** | **DEGs number in category** | **All genes number in category** | **P-value** | **Pathway ID** |
| --- | --- | --- | --- | --- |
| **UP-REGULATED** | | | | |
| Plant-pathogen interaction | 91 | 314 | 4.85E-34 | ko04626 |
| Starch and sucrose metabolism | 54 | 318 | 2.55E-10 | ko00500 |
| Amino sugar and nucleotide sugar metabolism | 48 | 260 | 2.80E-10 | ko00520 |
| Plant hormone signal transduction | 46 | 323 | 1.27E-06 | ko04075 |
| alpha-Linolenic acid metabolism | 25 | 131 | 4.32E-06 | ko00592 |
| Phenylpropanoid biosynthesis | 37 | 242 | 6.97E-06 | ko00940 |
| Sulfur metabolism | 16 | 68 | 1.56E-05 | ko00920 |
| Ether lipid metabolism | 14 | 62 | 5.75E-05 | ko00565 |
| Phosphatidylinositol signaling system | 17 | 95 | 1.11E-04 | ko04070 |
| Glycerophospholipid metabolism | 22 | 143 | 2.24E-04 | ko00564 |
| Cysteine and methionine metabolism | 23 | 153 | 3.80E-04 | ko00270 |
| Endocytosis | 38 | 327 | 1.21E-03 | ko04144 |
| Linoleic acid metabolism | 12 | 67 | 2.50E-03 | ko00591 |
| Ascorbate and aldarate metabolism | 11 | 62 | 2.96E-03 | ko00053 |
| Flavonoid biosynthesis | 8 | 36 | 3.76E-03 | ko00941 |
| Pentose and glucuronate interconversions | 14 | 91 | 3.80E-03 | ko00040 |
| Carotenoid biosynthesis | 8 | 43 | 8.42E-03 | ko00906 |
| Biosynthesis of unsaturated fatty acids | 9 | 54 | 1.13E-02 | ko01040 |
| Nicotinate and nicotinamide metabolism | 7 | 41 | 1.97E-02 | ko00760 |
| Stilbenoid, diarylheptanoid and gingerol biosynthesis | 7 | 44 | 3.32E-02 | ko00945 |
| Inositol phosphate metabolism | 10 | 82 | 3.92E-02 | ko00562 |
| Zeatin biosynthesis | 3 | 12 | 4.35E-02 | ko00908 |
| **DOWN-REGULATED** | | | | |
| Photosynthesis - antenna proteins | 15 | 59 | 6.51E-10 | ko00196 |
| Thiamine metabolism | 8 | 25 | 3.53E-07 | ko00730 |
| Photosynthesis | 12 | 83 | 1.57E-05 | ko00195 |
| Carotenoid biosynthesis | 8 | 43 | 2.58E-05 | ko00906 |
| Linoleic acid metabolism | 10 | 67 | 3.41E-05 | ko00591 |
| Regulation of autophagy | 7 | 54 | 9.84E-04 | ko04140 |
| Stilbenoid, diarylheptanoid and gingerol biosynthesis | 6 | 44 | 1.83E-03 | ko00945 |
| alpha-Linolenic acid metabolism | 11 | 131 | 2.11E-03 | ko00592 |
| Vitamin B6 metabolism | 6 | 52 | 5.55E-03 | ko00750 |
| Flavonoid biosynthesis | 5 | 36 | 5.72E-03 | ko00941 |
| Taurine and hypotaurine metabolism | 2 | 7 | 9.99E-03 | ko00430 |
| Terpenoid backbone biosynthesis | 9 | 120 | 1.23E-02 | ko00900 |
| Glycine, serine and threonine metabolism | 10 | 150 | 1.25E-02 | ko00260 |
| Indole alkaloid biosynthesis | 1 | 1 | 2.43E-02 | ko00901 |
| Arginine and proline metabolism | 5 | 68 | 3.22E-02 | ko00330 |
| Glycerolipid metabolism | 7 | 114 | 4.53E-02 | ko00561 |
| Biosynthesis of amino acids | 21 | 484 | 4.90E-02 | ko01230 |
