# Supplementary material for: New insights into the role of MADS-box transcription factor gene CmANR1 on root and shoot development in chrysanthemum (Chrysanthemum morifolium)
Source: BMC Plant Biol. 2021 Feb 6;21:79. doi: 10.1186/s12870-021-02860-7 (PMC7866475; doi:10.1186/s12870-021-02860-7)
Supplement: Supplementary file 5 — Additional file 5: Table S2. The primers used in this study. [file 12870_2021_2860_MOESM5_ESM.docx]

**Table S2** Primers used in this study.

Primer name Sequence (5’ to 3’)

TR3302|c0_g2(LBD37)-F CTCTTCTGTCCCCGAGCACC

TR3302|c0_g2(LBD37)-R GTCCGACTGCTCCGTTTACTG

TR17652|c0_g1(GATA23)-F CGGTGGTTCAAGAAGCTGTACGT

TR17652|c0_g1(GATA23)-R CAATCCTCTGAGTTGTCCTCTTCG

TR19268|c0_g2(NLP6)-F GAGTTCCCTTTGGTCCACTATGC

TR19268|c0_g2(NLP6)-R AACTCTAAAACATACTCAGCATCGC

TR21309|c2_g1 (ERF3)-F TCACCTACGACACCATCGCTAA

TR21309|c2_g1 (ERF3)-R GCCTAGCCATCTTCGAGTTTTCT

TR19992|c0_g1(IAA10)-F ATCAATAATCGTCATCTATCCGTT

TR19992|c0_g1(IAA10)-R CCAAGGTCTGTGCTAACATTTCAT

TR13277|c0_g1(NAC1)-F AAAGAACCCTAAACCCTGAACCG

TR13277|c0_g1(NAC1)-R GTGAAATCTAAATCCAGGAGGCAAC

TR16462|c0_g2(LBD29)-F GCACCTTATTTCCCATCCGAC

TR16462|c0_g2(LBD29)-R TGCATCCGCTCTTTGATCGAC

TR1244|c2_g4(GLABRA 2)-F CTACCTGATGGGGTGGAAACAAG

TR1244|c2_g4(GLABRA 2)-R GTCGGGGAGTCACTTGTAAGAATCT

TR18879|c1_g1(ILR1)-F GGTGACAATGGGAGGTGAAGAT

TR18879|c1_g1(ILR1)-R AATAAGGCGAGTGCAAATGCT

CmUBI-F CTAATGAATGCTTACTGTGACCGAC

CmUBI-R
